# Feasibility of a community‐based delivery model for HIV pre‐exposure prophylaxis among bar patrons in rural South Africa

**DOI:** 10.1002/jia2.25848

**Published:** 2021-11-26

**Authors:** Megan A. Grammatico, Anthony P. Moll, Koeun Choi, Sandra A. Springer, Sheela V. Shenoi

**Affiliations:** ^1^ University of Connecticut School of Medicine Farmington Connecticut USA; ^2^ Church of Scotland Hospital Tugela Ferry South Africa; ^3^ Yale Internal Medicine Yale School of Medicine New Haven Connecticut USA; ^4^ Yale AIDS Program Section of Infectious Diseases Yale School of Medicine, Department of Internal Medicine New Haven Connecticut USA

**Keywords:** alcohol use disorder, differentiated service delivery, heterosexual, HIV prevention, PrEP, young men

## Abstract

**Introduction:**

South Africa, home to the world's largest HIV epidemic, has made great strides in improving access to HIV services, but specific groups, particularly young men, remain difficult to engage in the HIV care cascade. Alcohol use disorder, prevalent in South Africa, further complicates engagement. Congregate settings where alcohol is served, known as shebeens, are an ideal place to engage young people for HIV testing, treatment and prevention, including pre‐exposure prophylaxis (PrEP). Here, we characterize the uptake of PrEP in shebeen patrons and explore the effect of alcohol consumption on PrEP uptake by piloting a community‐based delivery model.

**Methods:**

In the rural Kwazulu‐Natal province (KZN) of South Africa, a field team made up of all men offered screenings outside of shebeens at 27 events over 6 months in 2020. Screenings included rapid HIV testing and Alcohol Use Disorder Identification Test (AUDIT). Participants who tested negative for HIV were offered PrEP as once daily oral tenofovir disoproxil fumarate/emtricitabine. Short‐term retention was determined. Logistic regression was performed to identify predictors of PrEP uptake, including unadjusted and adjusted odds ratios (OR) with 95% confidence interval.

**Results:**

One hundred and sixty‐two shebeen patrons were screened, and 136 (84%) were eligible for PrEP. Among those eligible, 37 (27%) completed clinical evaluation and initiated PrEP. Among PrEP initiators, 91.9% were men, median age was 26.0 years (interquartile range 21–31), 32.4% were employed, 18.9% had running water and 70.3% had AUDIT scores indicating hazardous drinking. Among 37 initiators, 25 (68%) were retained at 1 month, and 19 (51%) were retained at 4 months. Independent predictors of PrEP uptake among all bar patrons, and only men (108 screened and 34 initiators), included younger age (OR 0.92 [0.88–0.97]) and lifetime number of sexual partners (OR 1.07 [1.02–1.13]).

**Conclusions:**

Community‐based PrEP delivery after engagement at shebeens in rural South Africa is a feasible and novel approach to reach a traditionally difficult‐to‐engage population, particularly young men. In this small sample, sexual risk behaviours predicted PrEP uptake. Hazardous drinking was not a barrier to PrEP initiation.

## 1 INTRODUCTION

While access to testing and antiretroviral therapy (ART) have vastly improved, young people, particularly men, remain difficult to engage in HIV care and prevention [1]. Men in South Africa have an estimated 10‐year gap in life expectancy compared to women [2]. Traditional cultural and gender norms, migratory work and dissatisfaction with healthcare all influence men being less likely than women to undergo HIV testing, initiate treatment and be retained in care [3, 4].

Hazardous alcohol use from mild to severe alcohol use disorder (AUD) further complicates the engagement in HIV care [5]. While chronic alcohol users living with HIV may achieve durable viral suppression, alcohol misuse can impact ART adherence, decrease viral suppression, and increase risky sexual behaviours, escalating the risk of HIV transmission [5]. AUD prevalence in South Africa is 7%, compared to 3.7% in other sub‐Saharan African countries [6, 7], higher for men (12.4%) than for women (1.8%) and higher in rural than urban settings [8, 9]. Patrons at shebeens, congregate settings where alcohol is served, report high‐risk behaviours making these an ideal setting for engaging young men for HIV prevention and treatment [10, 11, 12]. Pre‐exposure prophylaxis (PrEP) is a highly efficacious biomedical HIV prevention tool [13]. The greatest gap in the PrEP cascade is uptake, particularly for young and mobile individuals [14]. Community‐based PrEP delivery has the potential to address this gap but few models exist, particularly in rural areas [3, 8, 14, 15, 16, 17, 18, 19]. Furthermore, little data exist on PrEP use in the context of alcohol consumption.

With PrEP implementation expanding globally, the ability to access hard‐to‐reach rural populations through community‐based engagement is of paramount importance. We speculated that engaging young men at shebeens may be a feasible and effective way of promoting PrEP use and that alcohol misuse would deter PrEP use. We aimed to characterize PrEP uptake among shebeen patrons at high risk for HIV, determine whether alcohol misuse impacted PrEP uptake, and describe retention in a community‐based PrEP delivery model in a rural South African setting.

## 2 METHODS

### 2.1 Study setting

This study was conducted in rural Kwazulu‐Natal (KZN) province, characterized by extreme poverty and high (27%) HIV prevalence [20]. Many households lack access to running water, and transportation is difficult due to unpaved roads [21].

### 2.2 Community screening procedures

Integrated communicable and non‐communicable disease screenings were conducted for 6 months in 2020 outside shebeens by a lay community health worker and enrolled nurse team comprised only of men. In an NGO‐labelled mobile clinic parked outside the shebeen, staff engaged community members prior to entering shebeens, offering voluntary health screening [22]. Those who endorsed prior alcohol that day were asked to reschedule. Screening required verbal consent and included tuberculosis symptom screen, automated blood pressure reading, random blood glucose, third‐generation rapid fingerstick point‐of‐care HIV antibody test and sexually transmitted infection (STI) symptom screen. Those screened also completed a risk assessment, including sexual risks, drug use and the Alcohol Use Disorder Identification Test (AUDIT) [23], with hazardous drinking defined as ≥6 for women and ≥8 for men [24]. Those with positive screening results were referred to local primary health clinics.

Permission was granted by shebeen proprietors prior to the study and confirmed on the day of screening. Screening was performed at 27 shebeens, each approximately 4 hours, with approximately 8–9 shebeen patrons approached per event.

### 2.3 Eligibility

Eligibility criteria for the study included being ≥18 years old, testing negative for HIV and meeting indications for PrEP according to local guidelines, including condomless sex in the last month, STI in the last 6 months, sex under the influence of alcohol or drugs, HIV‐positive partner or having a sexual partner of unknown HIV status [13]. Those identified as PrEP‐eligible were offered referral to the study or primary care clinics. Those interested in the study met with staff on a separate day and time at non‐shebeen locations to review study activities and, if interested, proceed with written informed consent.

### 2.4 Enrolment

The eligibility assessment occurred in the mobile clinic at a mutually agreed‐upon location, usually close to the participants’ home. After consent, HIV‐negative status was reconfirmed using point‐of‐care fourth‐generation testing, and venipuncture was performed for haemoglobin, creatinine, ALT and hepatitis B surface Ag [13]. All participants provided urine for a point‐of‐care dipstick toxicology test (Redwood Toxicology, Panel 5, COC/THC/MOP/m‐AMP/BZO RediTest) and women submitted urine for beta HCG.

Given under‐reporting of alcohol use in other settings [25], dried blood spots were obtained at enrolment for phosphatidylethanol (PEth) level, where clinically significant alcohol use was defined as PEth ≥20 ng/ml [23]. Participants were scheduled for a second community visit 1–2 days later in the mobile clinic, receiving test results and counselling, and initiating PrEP, defined as receiving the first 30‐day supply of tenofovir disoproxil fumarate/emtricitabine (TDF/FTC). Participants scheduled a follow‐up visit at the mobile clinic at 1 and 4 months, with subsequent transfer to primary care clinics to continue PrEP care.

### 2.5 Analysis

Descriptive statistics were performed and bivariate analysis assessed predictors of PrEP initiation. Between group comparisons used parametric or non‐parametric (continuous variables) or chi‐square (categorical) tests. Logistic regression identified independent predictors of PrEP uptake and generated unadjusted and adjusted odds ratios (OR) with 95% confidence intervals. Correlation between AUDIT and PEth scores was determined using Pearson's *r*. Data were analysed using IBM SPSS Statistics v. 26. The study was approved by the South African Medical Association and Yale University Human Investigations Committee.

## 3 RESULTS AND DISCUSSION

Over 6 months, 229 shebeen patrons were approached, 162 (70.7%) participated in community‐based screenings, and 136 (84.0%) met criteria for PrEP through the study [13]. Those who did not meet criteria tested positive for HIV (*n* = 20, 12%), had no reported risk factors for HIV (*n* = 2) or were <18 years (*n* = 4) and thus not eligible for the study and referred to primary care clinics offering PrEP. Among those living with HIV, four were newly diagnosed and linked to care at their local clinic, and 16 were already on ART by self‐report.

**Figure 1:**
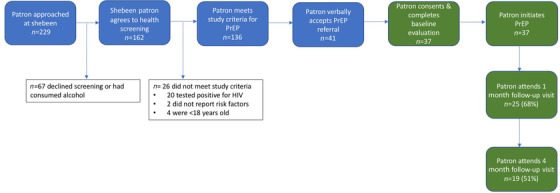
PrEP cascade among shebeen patrons. Blue boxes indicate the screening procedures that took place in the mobile clinic outside of the shebeen. Green boxes indicate PrEP procedures that were performed in the mobile clinic at a non‐shebeen community location.

Among those PrEP‐eligible (*n* = 136), the median age was 28 (interquartile range 23–40), 108 (79.4%) were men (Table 1) and 37 (27.2%) initiated PrEP (Figure 1). PrEP initiators (*n* = 37) did not differ significantly from non‐initiators (*n* = 99) on socio‐demographic characteristics, AUDIT score, or proportion with hazardous drinking. Younger age, being a man, greater number of sex partners in the last month and lifetime, and “never” having attended clinic all predicted PrEP uptake. Independent predictors included younger age (aOR 0.92 [0.88–0.97]) and greater number of lifetime sex partners (aOR 1.07 [1.02–1.13]). Among men only (*n* = 108, *n* = 34 initiators), the same independent factors predicted PrEP uptake (Table 2).

**Table 1:** Characteristics of bar patrons eligible for PrEP (*n* = 136)

Proportion or median (IQR)	All PrEP‐eligible (*n* = 136)	Non‐initiators (*n* = 99)	PrEP initiators (*n* = 37)	*p*‐Value	Men only (*n* = 108)	Men only non‐initiators (*n* = 74)	Men only PrEP initiators (*n* = 34)	*p*‐Value
Median age (IQR)^a^	28 (23–40)	30.0 (24–43)	26.0 (21‐31)	**0.035**	28 (23–38.8)	29 (24–41.3)	26 (21–31)	**0.06**
Men^b^	108 (79.4%)	74 (74.7%)	34 (91.9%)	**0.028**	–	–	–	–
Employed^b^	41 (30.1%)	29 (29.3%)	12 (32.4%)	0.72	38 (35.2%)	26 (35.1%)	12 (35.3%)	0.58
Smoker (cigarettes)^b^	73 (53.7%)	53 (53.5%)	20 (54.0%)	0.96	71 (65.7%)	51 (68.9%)	20 (58.8%)	0.98
Marijuana user^b^	19 (14%)	12 (12.1%)	7 (18.9%)	0.3	18 (16.7%)	11 (14.9%)	7 (20.6%)	0.46
Median AUDIT score (IQR)^a^	10 (6–14.75)	10 (6–14)	11 (6.5–16)	0.46	11 (8–15)	11 (8–14)	11 (7–16.5)	0.86
Hazardous drinkers^b^	97 (71.3%)	71 (72.4%)	26 (70.3%)	0.8	87 (80.6%)	62 (83.8%)	25 (73.5%)	0.49
Inconsistent condom use^b^	125 (91.4%)	90 (91%)	35 (94.6%)	0.48	101 (93.5%)	69 (93.2%)	32 (94.1%)	0.8
History of STI^b^	8 (5.9%)	5 (5.05%)	3 (8.1%)	0.5	7 (6.5%)	4 (5.4%)	3 (8.8%)	0.39
Median number of sex partners in the last 1 month (IQR)^a^	1 (1–2)	1 (1–1)	1 (1–2)	**0.04**	1 (1–2)	1 (1–1.25)	1 (1–2)	0.11
Median number of sex partners in lifetime (IQR)^a^	9.5 (5–15)	8 (5–11)	12 (8.5–15)	**0.02**	10 (5.3–15)	10 (5–15)	12 (9–16.3)	**0.06**
Never attended clinic^b^	78 (57.4%)	51 (51.5%)	27 (73.0%)	**0.02**	66 (61.1%)	41 (55.4%)	25 (73.5%)	**0.07**

Abbreviations: AUDIT, Alcohol Use Disorder Identification Test; CI, confidence interval; IQR, interquartile range; STI, sexually transmitted infection.

*P*‐value <0.05 indicates statistical significance.

^a^ Mann–Whitney U.

^b^ Chi‐square.

**Table 2:** Predictors of PrEP uptake among bar patrons

	All shebeen patrons (*n* = 136)		Men only (*n* = 108)	
	Unadjusted odds ratio (95% CI)^a^	Adjusted odds ratio (95% CI)^a^	Unadjusted odds ratio (95% CI)^a^	Adjusted odds ratio (95% CI)^a^
Age (years)	0.93 (0.89–0.97)	0.92 (0.88–0.97)	0.93 (0.88–0.98)	0.93 (0.88–0.98)
Man	3.83 (1.1–13.6)			
Number of sex partners in the last month	1.6 (0.93–2.89)			
Number of sex partners in lifetime	1.07 (1.01–1.13)	1.07 (1.02–1.13)	1.05 (1.0–1.12)	1.05 (0.99–1.12)
Never attended clinic	2.54 (1.13–5.8)		1.77 (0.92–3.4)	1.13 (0.42–3.1)

^a^ Logistic regression.

Among PrEP initiators (*n* = 37), 25 (68%) and 11 (30%) were retained at 1 and 4 months, respectively (Figure 1), and were subsequently transferred to their local primary care clinic to continue PrEP. Predictors of loss to follow up were not identified, including AUDIT score, given the small sample size.

Among those PrEP‐eligible, 97 (71%) had AUDIT scores indicating hazardous drinking. Among 25 (68%) initiators with results available, 20 (80%) had a positive result (≥20 ng/ml); four (20%) had positive PEth results but did not meet AUDIT criteria for hazardous drinking. AUDIT and PEth result was significantly correlated (Pearson's *r* = 0.46, *p* = 0.02).

We evaluated a community‐based model of PrEP delivery targeting shebeen patrons in rural South Africa. Our findings indicate that PrEP delivery among this traditionally hard‐to‐reach population is both feasible and acceptable, and that sexual risk behaviours predicted PrEP uptake, while highly prevalent alcohol use did not predict PrEP uptake. Our previous work [26] has shown high uptake of HIV testing outside of shebeens, where a substantial proportion of young men are engaging in high‐risk behaviours [13]. These findings underscore the value of using shebeens as a strategy for reaching young men, who are central to HIV transmission [27].

In this study, a greater number of lifetime sex partners predicted PrEP uptake among all shebeen patrons and among only men, indicating that participants correctly perceive their sexual behaviours are high risk, and are interested in discreet mitigation strategies that do not require the knowledge of their partners or, as with condoms, that are perceived to interfere with sex [4]. Men's acceptance of PrEP has primarily been evaluated among men who have sex with men [28]. Men who have sex with women acknowledge that multiple sexual partners are risky but culturally acceptable, and that they would consider PrEP as an acceptable risk‐mitigation strategy, though anticipate barriers with clinic‐based care [18, 19].

Our model was focused on engagement at ubiquitous alcohol‐serving venues that support socializing. The community team composed of men may have positively influenced PrEP uptake in men, but may have dissuaded women. While only one in five of those HIV tested at shebeens were women, less than one in 10 who initiated PrEP were women – likely reflecting that shebeens in rural areas are traditionally attended by men but also possible discomfort with a screening team entirely comprised of men. The value of gender concordance of the screening team needs to be clarified. Nonetheless, these data suggest that a differentiated service delivery model to meet men where they are in the community is feasible and important to engaging men in HIV prevention. Participants overall, but in particular PrEP initiators, reported lack of engagement at the primary care clinics, supporting the utility of a community‐based approach for reaching young people frequenting shebeens and consuming alcohol who do not engage in care. Like men, different populations (i.e. serodiscordant couples, adolescent girls and young women) may be better served by an implementation strategy tailored to their unique needs [16].

A high proportion of patrons screened met AUDIT criteria for hazardous drinking, though this did not influence PrEP uptake or retention. Though a small sample, this is consistent with previous work linking shebeen attendance with HIV risk regardless of alcohol consumption, attributable to the shebeen environment itself influencing risky sexual behaviour [29]. Frequency, type and quantity of alcohol use may be more influential for adherence and retention in PrEP care. Despite restrictions imposed by the COVID‐19 pandemic and potential resulting changes in risk behaviour, short‐term retention in PrEP care was in line with other published data [30]. Since predictors of retention were not identified in our small study, larger studies to explore barriers to retention among young men and women who drink are NEEDED.

We recognize several limitations. First, we used a convenience sample, rather than a population‐based sample, limiting generalizability. Additionally, comparisons between patrons who initiated and did not initiate PrEP were limited due to small sample size, attributable to COVID‐19 pandemic‐related disruptions, including the repeated shutdown of all shebeens in South Africa [31, 32]. Additionally, pandemic‐related restrictions may have dissuaded individuals from continuing PrEP or resulted in decreased risk behaviours and decreased perceived risk. Next, PEth samples were available only for a subset of enrolees due to COVID‐19‐related decreased nursing availability. Nonetheless, the limited results suggest that PEth has fair concordance with AUDIT, suggesting that participants consistently reported alcohol consumption and that AUDIT may be a reliable tool in this population. PEth may be helpful in adjunctively evaluating the role of alcohol use in monitoring PrEP adherence and outcomes. Despite these limitations, this proof‐of‐concept study demonstrates the feasibility of engaging young people, particularly men, for PrEP at shebeens in rural settings. Significant associations despite the small sample are noteworthy, though validation in larger studies is required.

## 4 CONCLUSIONS

Community‐based PrEP delivery is a promising strategy for reaching young people who consume alcohol in rural South Africa. Sexual risk behaviours predicted PrEP uptake, supporting the potential for shebeen‐based engagement to influence PrEP implementation. Hazardous alcohol use was not a significant predictor of PrEP initiation in this feasibility study. Larger studies are required to explore the impact of community‐based models of PrEP delivery on PrEP outcomes.

## COMPETING INTERESTS

SAS has received honoraria from Alkermes Inc and received in kind Vivitrol for prior NIDA and NIAAA funded studies and Sublocade from Indivior Inc. She has also received honoraria from DKBmed and HIV Learning Lab via grants supported from Gilead and for CME lectures for PRIME via support from ViiV pharmaceutical company. SVS's spouse previously worked for Merck Pharmaceuticals 1997–2007 and retains stock in his retirement account. There is no conflict, but is included for full disclosure. All other authors report no competing interests.

## AUTHORS’ CONTRIBUTIONS

MG, APM, KC, SAS and SVS conceived of and designed the study. MG, APM and SVS collected the data. MG and SVS analysed the data. MG, APM, KC, SAS and SVS contributed to the writing and/or revising of the manuscript. All authors have read and approved the final manuscript.

## FUNDING

MG was supported by the NIH Fogarty Global Health Equity Scholar TW010540. KC was supported by the Doris Duke Charitable Foundation #2016178. SVS was supported by the Doris Duke Charitable Foundation, grant #2015216. SAS is supported by the National Institute on Drug Abuse (NIDA) K02DA032322.

## Data Availability

The data is available at DOI: 10.17632/3d95jhy5px.1
